# Cohort profile: investigating SARS-CoV-2 infection and the health and psychosocial impact of the COVID-19 pandemic in the Canadian CHILD Cohort

**DOI:** 10.4178/epih.e2023091

**Published:** 2023-10-13

**Authors:** Rilwan Azeez, Larisa Lotoski, Aimée Dubeau, Natalie Rodriguez, Myrtha E. Reyna, Tyler Freitas, Stephanie Goguen, Maria Medeleanu, Geoffrey L. Winsor, Fiona S. L. Brinkman, Emily E. Cameron, Leslie Roos, Elinor Simons, Theo J. Moraes, Piush J. Mandhane, Stuart E. Turvey, Shelly Bolotin, Kim Wright, Deborah McNeil, David M. Patrick, Jared Bullard, Marc-André Langlois, Corey R. Arnold, Yannick Galipeau, Martin Pelchat, Natasha Doucas, Padmaja Subbarao, Meghan B. Azad

**Affiliations:** 1 Department of Immunology, University of Manitoba, Winnipeg, MB, Canada; 2 Children’s Hospital Research Institute of Manitoba, Winnipeg, MB, Canada; 3 Department of Pediatrics and Child Health, University of Manitoba, Winnipeg, MB, Canada; 4 Division of Respiratory Medicine, Department of Pediatrics and Program in Translational Medicine, SickKids Research Institute, The Hospital for Sick Children, Toronto, ON, Canada; 5 Department of Medicine, Faculty of Health Sciences, McMaster University, Hamilton, ON, Canada; 6 Department of Molecular Biology and Biochemistry, Simon Fraser University, Burnaby, BC, Canada; 7 Department of Psychology, University of Manitoba, Winnipeg, MB, Canada; 8 Department of Pediatrics, University of Alberta, Edmonton, AB, Canada; 9 Department of Pediatrics, University of British Columbia, Vancouver, BC, Canada; 10 Centre for Vaccine Preventable Diseases, University of Toronto; Dalla Lana School of Public Health, University of Toronto; Department of Laboratory Medicine and Pathobiology, University of Toronto; Public Health Ontario, ON, Canada; 11 Department of Community Health Sciences, Cumming School of Medicine, University of Calgary, Calgary, AB, Canada; 12 Strategic Clinical Networks, Alberta Health Services, Calgary, AB, Canada; 13 School of Population and Public Health, University of British Columbia, Vancouver, BC, Canada; 14 British Columbia Centre for Disease Control, Vancouver, BC, Canada; 15 Cadham Provincial Laboratory, Manitoba Health, Winnipeg, MB, Canada; 16 Department of Biochemistry, Microbiology and Immunology, University of Ottawa, Ottawa, ON, Canada; 17 CHILD Cohort Study National Parent Engagement Committee

**Keywords:** COVID-19, SARS-CoV-2, Immunology, Epidemiology, Pediatrics, Cohort studies

## Abstract

The coronavirus disease 2019 (COVID-19) pandemic has affected all Canadian families, with some impacted differently than others. Our study aims to: (1) determine the prevalence and transmission of severe acute respiratory syndrome coronavirus 2 (SARS-CoV-2) infection among Canadian families, (2) identify predictors of infection susceptibility and severity of SARS-CoV-2, and (3) identify health and psychosocial impacts of the COVID-19 pandemic. This study builds upon the CHILD Cohort Study, an ongoing multi-ethnic general population prospective cohort consisting of 3,454 Canadian families with children born in Vancouver, Edmonton, Manitoba, and Toronto between 2009 and 2012. During the pandemic, CHILD households were invited to participate in the CHILD COVID-19 Add-On Study involving: (1) brief biweekly surveys about COVID-19 symptoms and testing; (2) quarterly questionnaires assessing COVID-19 exposure and testing, vaccination status, physical and mental health, and pandemic-driven life changes; and (3) in-home biological sampling kits to collect blood and stool. In total, 1,462 households (5,378 participants) consented to the CHILD COVID-19 Add-On Study: 2,803 children (mean±standard deviation [SD], 9.0±2.7 years; range, 0-17 years) and 2,576 adults (mean±SD, 43.0±6.5 years; range, 18-85 years). We will leverage the wealth of pre-pandemic CHILD data to identify risk and resilience factors for susceptibility and severity to the direct and indirect pandemic effects. Our short-term findings will inform key stakeholders and knowledge users to shape current and future pandemic responses. Additionally, this study provides a unique resource to study the long-term impacts of the pandemic as the CHILD Cohort Study continues.

## INTRODUCTION

Severe acute respiratory syndrome coronavirus 2 (SARS-CoV-2) infection results in a broad range of clinical phenotypes, from asymptomatic infection to severe disease and death [1,2]. Pre-existing medical conditions, socioeconomic disadvantage, occupation, gender, and race/ethnicity have been associated with SARS-CoV-2 infection rates and disease severity in adults [3-6]. However, much of the biological variation in this new disease remains unexplained, and more research is needed to understand why children are less likely than adults to develop severe coronavirus disease 2019 (COVID-19) [7-9]. Identifying these divergent outcomes in adults and children will help inform prevention and treatment strategies for people of all ages [10]. Additionally, understanding the persistence and biology of antibody responses following infection or vaccination will help inform public health responses to the ongoing pandemic.

In an effort to control this pandemic, public health measures have varied across Canada both geographically and over time. COVID-19 vaccination and non-pharmaceutical interventions, such as masking and school closures, have helped slow the spread of COVID-19 in Canada and beyond [3,11,12]. However, these measures have had consequences for the economy, mental health, and emotional and physical well-being of family members. They may also amplify inequities experienced by marginalized and vulnerable populations [12]. It is therefore important to assess how pandemic control measures differentially affect the short-term and long-term physical and mental health of Canadian families.

**Figure 1.**
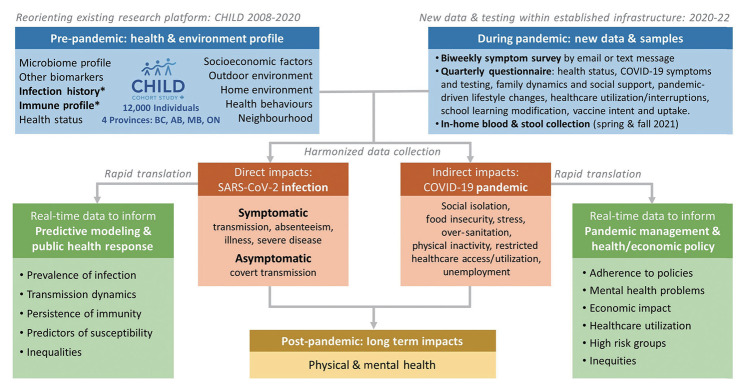
Overview of the CHILD COVID-19 Add-On Study. The CHILD COVID-19 Add-On Study, a sub-study of the CHILD study, was developed in response to the COVID-19 pandemic. The CHILD COVID-19 Add-On Study will leverage the rich pre-pandemic CHILD dataset with the newly collected pandemic dataset to study the direct effects of SARS-CoV-2 infection and the indirect effects of the COVID-19 pandemic among study participants and their households across 4 provinces in Canada. Data will be collected for the CHILD COVID-19 Add-On Study through (1) biweekly surveys to assess COVID-19 symptoms and testing; (2) quarterly questionnaires on health care utilization, lifestyle, employment status, mental and physical health during the pandemic; and (3) in-home stool and finger-prick blood samples. CHILD, Canadian Healthy Infant Longitudinal Development; COVID-19, coronavirus disease 2019; SARS-CoV-2, severe acute respiratory syndrome coronavirus 2; BC, British Columbia; AB, Alberta; MB, Manitoba; ON, Ontario.

The CHILD Cohort Study (www.childstudy.ca), an ongoing birth cohort of 3,454 families, offers a unique opportunity to study COVID-19, as the CHILD Cohort Study has collected a wealth of socio-demographic, health, and biological data since 2009. The CHILD COVID-19 Add-On Study (Figure 1) will investigate (1) symptomatic and asymptomatic SARS-CoV-2 infection prevalence, transmission, and immunity; (2) predictors of SARS-CoV-2 infection susceptibility and severity; (3) prevalence and predictors of health and psychosocial impacts of the COVID-19 pandemic on CHILD families; and (4) individual and social determinants of COVID-19 vaccine uptake or hesitancy. Furthermore, it will provide a lasting resource to study the long-term impacts of the pandemic as the CHILD Cohort Study continues.

## COHORT DESCRIPTION

### Study design and population

**Figure 2.**
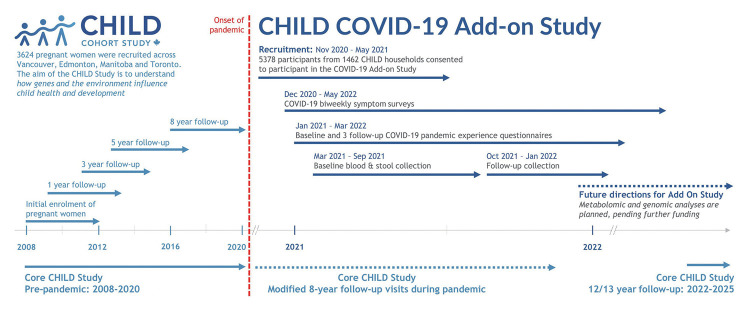
Timeline for the core CHILD Cohort Study and CHILD-COVID-19 Add-On Study. The CHILD Cohort Study, launched in 2008, is an ongoing prospective, population-based cohort study involving 3,454 families recruited from Vancouver, Edmonton, Manitoba, and Toronto. The CHILD COVID-19 Add-On is embedded in the CHILD Study and was designed in 2020 to understand how the pandemic affects CHILD families. CHILD, Canadian Healthy Infant Longitudinal Development; COVID-19, coronavirus disease 2019.

This is a prospective longitudinal study embedded in the existing CHILD cohort, which recruited 3,454 families (~10,000 individuals) from the general population with children born in British Columbia, Alberta, Manitoba, and Ontario between 2009 and 2012 (90.7% retention to date) [13]. The eligible participants for the COVID Add-On Study included all parents and children enrolled in the CHILD Cohort Study, along with all other individuals living in the same household (n=3,032 eligible households) (Figure 2). This COVID Add-On Study involves (1) brief biweekly surveys about COVID-19 symptoms and testing; (2) quarterly questionnaires assessing COVID-19 exposure and vaccination status and pandemic-driven life changes; (3) in-home collection of blood for SARS-CoV-2 immunoglobulin G serology, biomarker analysis, and (optionally) genetic analysis, and stool for microbiome analysis.

### Consent, recruitment, and retention

Eligible participants were invited to take part in this study during in-person CHILD Cohort Study clinic visits or by phone, video call, or e-mail, between November 2020 and May 2021. E-mail invitations included a summary of the study, a YouTube video (https://www.youtube.com/watch?v=lEzbn7HYiQw) describing the study’s purpose and participation requirements, and instructions on how to arrange a virtual consent appointment with research staff. Participants attending clinic visits were provided paper consent forms. Otherwise, consent was obtained virtually during a videoconference appointment with study staff (Zoom; Zoom Video Communications, Inc., San Jose, CA, USA) using the web browser-based Research Electronic Data Capture (REDCap) consent module [14,15]. Child participant assent (as defined provincially by age) or consent was obtained in the same manner in the presence of their caregiver. Participants were invited to additionally provide consent for 3 optional activities: (1) whole genome sequencing (65.1% consented), (2) future undefined analyses of their biological samples (69.4%), and (3) analysis of previously collected biological samples for future unknown research studies (59.4%). To encourage retention, we provided gift cards (US$25/household for completion of the baseline survey, and again for return of biological samples) and returned SARS-CoV-2 serology results to participants.

### Biweekly surveys

Study participants’ COVID-19 symptoms, exposure and diagnosis were captured through a biweekly symptom survey. Beginning in December 2020, 1 designated participant per household was prompted biweekly by text message or e-mail (according to their preference) with 3 COVID-19 screening questions asking if anyone in their household had symptoms of, was suspected of having, or was tested for COVID-19. Participants responding “yes” to any of these questions were sent a follow-up COVID-19 symptom survey by e-mail to collect individual-level information on specific COVID-19 symptoms and testing, travel, and exposure history, as well as clinical or public health action taken after a positive COVID-19 test result. Completion rates for the biweekly surveys ranged from 70.0% in the Edmonton site to 88% in the Winnipeg site and were generally consistent over time.

### Quarterly questionnaires

Questionnaires (Supplementary Material 1) were delivered online using REDCap at enrolment and approximately every 3 months thereafter. They were adapted from other Canadian COVID-19 studies, and addressed the following topics: individual demographic characteristics, employment status, COVID-19 pandemic government support use, mental and physical health, school closures, adherence to non-pharmaceutical public health measures, screen time and use of technology for school/work or other purposes, COVID-19 symptoms, testing and diagnosis, travel history and COVID-19 vaccination perceptions and status (Table 1). There were 3 versions of each questionnaire, designed for completion by children (about themselves), adults (about themselves), or parents/caregivers (about their children). Individuals able to provide consent on their own behalf were asked to complete the adult questionnaire. All remaining participants (i.e., children enrolled through assent or caregiver consent) were asked to complete the child questionnaire. The caregivers of participating children were also asked to complete a parent questionnaire for each child enrolled in their household. The completion rates for the quarterly questionnaires ranged from 84.9% (n=6,287) at baseline to 43.9% (n=3,248) at the final follow-up (Table 2). In general, the socio-demographic characteristics of participants who completed the final follow-up survey (51.3% of adults and 49.0% of children) did not differ much from those who did not. However, adult participants who did not complete the final survey were slightly more likely to have a lower level of formal education (Supplementary Material 2).

### In-home biological sample collection and severe acute respiratory syndrome coronavirus 2 serology

Blood and stool samples were collected twice (spring and fall/winter 2021) using in-home sampling kits sent to each household along with a pre-paid return shipment box. Participants were asked to collect all samples within a 48-hour period of receipt. Sample kits were prepared by the DaklaPack company (Moonachie, NJ, USA) and included all materials required to collect blood and stool, including an instructional pamphlet (Supplementary Material 3), 2-ply 70% alcohol swabs, 2”× 2” 8-ply sterile gauze sponges (CA95041-740; VWR, Radnor, PA, USA), bandages, a registration card and specimen bag containing a Desco Humidity Indicator Cards (89131-360; VWR International) and MiniPax absorbent desiccant packets (Z163570; Sigma-Aldrich, St. Louis, MO, USA). Sample packages were returned to the Clinical Research Laboratory and Biobank (CRLB, Hamilton, ON, Canada) by airmail. The collection rates for biological samples were 56.4% in the first wave and 57.0% in the second wave of collection (Table 2).

#### Blood collection

Blood specimens were collected using the dried blood spot (DBS) method and Mitra Blood Collection devices. DBS samples were collected using a Whatman 903 protein saver 5-spot card (VWR International). Returned sample bags containing DBS collection cards, desiccant pouches, and humidity cards were sealed in a second polybag and stored at 4°C for SARS-CoV-2 serological analysis using automated chemiluminescent enzyme-linked immunosorbent assays to detect anti-spike and anti-nucleoprotein antibodies as described by Cholette et al. [16]. COVID-19 antigens and the anti-hIgG#5-HRP fusion antibody were generously provided by Dr. Yves Durocher, National Research Council of Canada. The cut-offs for seropositivity used were based on false discovery rates in pre-pandemic cohorts of 2% for the spike and both 1% and 10% for the nucleoprotein. Preserved blood was also collected using Mitra clamshell devices containing 2 samplers (Neoteryx, Torrance, CA, USA). Upon receipt, the clamshell casing was removed, samplers were loaded into a 96-autorack, placed into a polybag containing a desiccant pouch, and stored at -80°C until further use. Metabolomic and genomic analyses are planned, pending further funding.

#### Stool collection

Stool samples were collected by participants using 2 cottontipped swabs. Fecal swabs were then suspended in a capped collection tube containing ethanol for transport. Upon receipt, the shafts of the fecal swabs were cut off and the specimens were cryopreserved. Microbiome analyses are planned, pending further funding.

### Statistical analysis plan

#### Aim 1: estimate SARS-CoV-2 infection, transmission, severity, and immunity

The prevalence and severity of infection will be estimated from self-reported biweekly reports, quarterly questionnaire testing information, and serological testing. Severity will be classified as asymptomatic, mild, or severe using World Health Organization criteria [17]. Transmission will be assessed using multilevel modelling (by household and study site). Persistence of SARS-CoV-2-induced immunity will be determined from longitudinal serology testing for SARS-CoV-2 antibodies over 2 time frames (spring and fall 2021).

#### Aim 2: identify predictors of SARS-CoV-2 infection susceptibility and severity

We will combine new data from the CHILD COVID-19 Add-On Study with existing pre-pandemic CHILD data to predict SARS-CoV-2 infection susceptibility (incidence) and severity. We hypothesize that infection susceptibility will be associated with (1) physical interactions with (or distancing from) other individuals in the community during the pandemic; (2) socio-demographic characteristics including sex, age, occupation, socioeconomic status, and household size; (3) pre-existing medical conditions including obesity and asthma; (4) neighbourhood characteristics including air pollution exposure, housing type, green and blue spaces; and (5) pre-pandemic immune biomarker and genetic profiles, viral infection history (including pre-existing coronavirus serology), respiratory health (wheezing, lung function), and health-related lifestyle factors (e.g., diet quality and physical activity). Associations will be tested in multivariate regression models with a 3-category outcome (symptomatic, asymptomatic, or no infection), random forest analysis, gradient boosting, recursive feature elimination and mutual information method. If sufficient cases are identified, we will also evaluate infection severity.

#### Aim 3: understand the impact of the COVID-19 pandemic on the mental and physical health of Canadian families using a health equity lens

We will quantify the prevalence of and changes between prepandemic and during-pandemic physical and mental health functioning (not directly related to SARS-CoV-2 infection). Prediction models, as described for aim 2, will be used to identify factors that predict risk for (or protection against) poor pandemic-related mental and physical health functioning. We hypothesize that protective factors will include (1) maintaining social connections, physical activity, healthy eating behaviours, and adequate sleep during the pandemic; (2) higher pre-pandemic mental well-being; and (3) continued employment throughout the pandemic.

### Ethics statement

This study was approved by research ethics boards at the University of British Columbia (H20-02324), University of Alberta (Pro00102524), University of Manitoba (HS24250), The Hospital for Sick Children (1000071220), and McMaster University (1108).

## KEY FINDINGS

**Figure 3.**
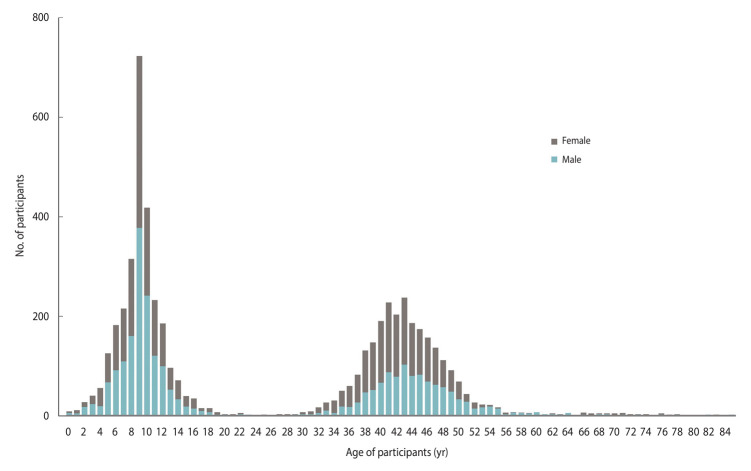
Age and sex distribution of CHILD-COVID-19 Add-On Study participants. CHILD, Canadian Healthy Infant Longitudinal Development; COVID-19, coronavirus disease 2019.

The final CHILD COVID-19 Add-On Study population included 5,378 participants from 1,462 households (mean, 3.7 participants per household; interquartile range [IQR], 3-4; range, 1-12), of whom 3,848 were original CHILD participants (1,431 mothers, 1,015 fathers, and 1,402 children). The remaining 1,530 participants were siblings (n = 1,427) or other household members (n=103). Almost half (48%) of the eligible CHILD households consented to participate in the CHILD COVID-19 Add-On Study. A total of 370 households (12% of those eligible) actively declined to participate, while the remainder were unresponsive (40%, n=1,205). Compared to non-participating CHILD families, those participating in the COVID-19 Add-On Study tended to have higher education and household income, and larger households (Supplementary Material 4). Participants were distributed across the Manitoba (34%), Vancouver (27%), Toronto (22%), and Edmonton (17%) study sites. Among children enrolled (n=2,802, 52% female), the mean age was 9.0 years (standard deviation [SD], 2.7; range, 0-17), while among adults (n = 2,576, 58% female), the mean age was 43.0 years (SD, 6.5; range, 18-85) (Figure 3). Participants predominantly identified as having European ancestral origins (77%), and the majority (68%) of adults had a university degree.

At enrolment, 31% of adults were essential workers (e.g., healthcare, delivery, store, security, and building maintenance), with proportions ranging from 17% in Toronto to 44% in Manitoba. During the first wave of the pandemic, 45% of working adults moved to remote work, 9% lost their job (permanently or temporarily), and 35% accessed government supports (e.g., loan deferrals, personal or business income support). The majority of children (84%) experienced school closures during spring 2020 (range, 80-89% across study sites), of which 23% returned to in-person classes before the end of the academic year in June 2020 (ranging from 6% in Edmonton to 51% in Vancouver). Most children who experienced school closures were offered (92%) and attended (85% fully and 13% partially) online classes (Table 3).

### Patient and public involvement statement

The CHILD COVID-19 Add-On Study adopts an integrated knowledge translation approach. To accelerate the availability and translation of high-quality, real-time evidence, we included key stakeholders on our team to develop the study and translate and disseminate results. Our team includes members of the CHILD Parent Advisory Committee and Knowledge Users from public health authorities at the national (Public Health Agency of Canada) and provincial levels (BC Centre for Disease Control, Alberta Health Services, Manitoba Shared Health, Public Health Ontario) who meet monthly with study investigators to share data in real time and adapt procedures if necessary (e.g., modify symptom surveys, questionnaires, and serology testing methods) to ensure that our results can be rapidly translated into policy and practice. The study investigators also meet monthly with the Canadian Covid Immunity Task Force Pediatric Working Group to share experiences and expertise, develop harmonized protocols, and foster research collaborations. A Rapid Results website (https://childstudy.ca/covid-rapid-results/) was developed to facilitate rapid widespread translation of study results to participants and stakeholders. To maximize recruitment, retention, and relevance to families, the CHILD Parent Advisory Committee was engaged to co-develop the study design, grant application, recruitment strategies, and questionnaires.

In addition to traditional dissemination of results through open access publication in peer-reviewed journals, we will use our website (childstudy.ca/covid-rapid-results) and social media (Twitter) to rapidly disseminate time-sensitive findings that are relevant to stakeholders and knowledge users, including provincial public health organizations and non-profit organizations (e.g., Children First Canada). We will also engage the CHILD Knowledge Mobilization Stakeholder Advisory Committee, which includes stakeholders from government, industry, patient organizations, clinical societies, child health-focused organizations, parenting-focused communications platforms, and CHILD parents.

## STRENGTHS AND WEAKNESSES

Our cohort offers a unique opportunity to study the clinical features and long-term sequelae of COVID-19, assess viral transmission within households, understand COVID-19 vaccine uptake/hesitancy, and investigate the psychosocial impact of the pandemic and its management among Canadian children and their families. The CHILD cohort has rich pre-pandemic longitudinal data and biosamples available to investigate how current and pre-pandemic health status, immune phenotypes and biomarkers can interactively predict susceptibility and resilience to COVID-19 and the psychosocial impacts of the pandemic.

**Figure 4.**
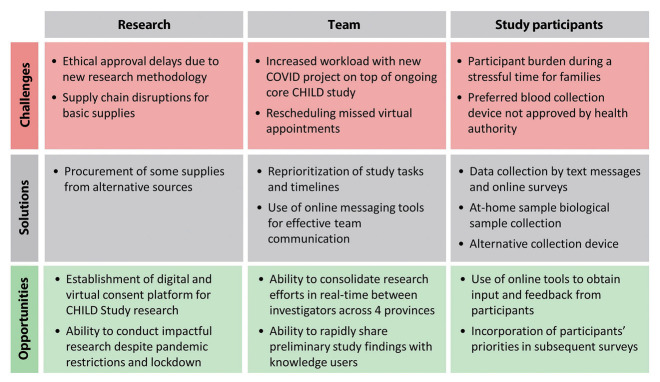
CHILD COVID-19 Add-On Study pandemic-related research challenges, solutions and opportunities. CHILD, Canadian Healthy Infant Longitudinal Development; COVID-19, coronavirus disease 2019.

Unanticipated challenges encountered during the pandemic delayed data collection during a critical period of the pandemic (summer 2020). For example, ethical approvals were delayed by the introduction of many novel study elements (e.g., virtual consent by video conference and in-home sample collection). Pandemic-driven supply chain disruptions made acquiring biological sample kit components (e.g., gauze and bandages) exceedingly difficult, leading to delays in sample collection. Despite being used in the United States for serology testing, the Mitra Clamshell devices were not approved by Health Canada in time for study deployment. Standard DBS methods were used instead, requiring a more painful finger prick and larger blood volume, which negatively impacted retention (Figure 4).

Generalizability may be limited by selection bias because socioeconomic status tended to be higher among CHILD families than in the general Canadian population. Further, we noted significant differences in socio-demographic characteristics (e.g., parental education and total household income) between participating and non-participating CHILD households. This selection bias may have excluded the most vulnerable populations during the precarious early pandemic period, further exacerbating the lack of generalizability of this cohort. Sensitivity analyses may be conducted to explore the robustness of study findings where applicable. The frequent biweekly questionnaires and lengthy adult and parent quarterly questionnaires created a large burden on the designated household participant responsible for providing responses for themselves and all household members. Collectively, the high burden placed on participants led to study withdrawal in a subset of families.

To our knowledge, there is no other multi-site pediatric cohort with rich longitudinal data and recent pre-pandemic biosamples available to investigate how current and pre-pandemic health status, immune phenotypes, and biomarkers can interaactively predict susceptibility and resilience to COVID-19 and the psychosocial impacts of the pandemic. The CHILD Cohort offers a unique and powerful opportunity to study the clinical features and longterm sequelae of COVID-19, assess viral transmission within households, understand COVID-19 vaccine uptake/hesitancy, and investigate the psychosocial impact of the pandemic and its management among Canadian children and their families.

## DATA ACCESSIBILITY

Data are available upon reasonable request. Researchers interested in developing or collaborating on a project using CHILD data are encouraged to contact the study’s National Coordinating Centre for a formal request. Details for data request and access are outlined on the CHILD website: https://childstudy.ca/for-researchers/study-data/.

## Figures and Tables

**Table 1.** CHILD COVID-19 Add-On Study questionnaires

Questionnaire domains & contents	Questionnaire & date of data collection				
	Baseline	Follow-up 1	Follow-up 2	Follow-up 3	Biweekly
	Jan-Jun, 2021	Aug-Sep, 2021	Oct-Dec, 2021	Jan-Mar, 2022	
COVID-19 exposure/health status					
Physical and mental health	x	x	x	x	-
COVID-19 exposure, symptoms, and testing	x	x	x	x	x
COVID-19 diagnosis and outcomes	x	x	x	x	x
Post-COVID (long-COVID) symptoms	-	-	-	x	-
Healthcare access and pandemic driven lifestyle changes					
Impact on healthcare access & delivery	x	x	x	x	-
Impact on travel, work, and childcare	x	x	x	x	-
Adherence to non pharmaceutical public health measures	x	x	x	x	-
Financial stability during the pandemic	x	x	x	x	-
Eating habit, sleep and physical activity	x	x	x	x	-
Government income support/benefits	x	x	x	-	-
Family and friends’ connection/relationship	x	x	x	-	-
Screen time and social media use	x	x	x	-	-
Alcohol and substance use during the pandemic	x	x	x	-	-
Disruptions and modifications to school learning	x	x	-	-	-
Family routines and dynamics	-	x	x	-	-
Perceptions towards returning to in-person learning	-	-	x	-	-
COVID-19 perceptions					
Pandemic induced worry, stress, anxiety, and loneliness	x	x	x	x	-
Stress and coping during the pandemic	x	x	x	x	-
Pandemic driven positive changes	x	x	x	-	-
Access to, and usage of mental health services	-	x	x	x	-
Social support during the pandemic	-	x	x	-	-
Demographic information					
Family and household structure	x	x	x	x	-
Occupation	x	x	x	-	-
Essential worker in household	x	x	x	-	-
Geographic ancestral origins	x	-	-	-	-
Level of formal education	x	-	-	-	-
Vaccination & medical history					
COVID-19 and flu vaccine uptake	x	x	x	x	-
COVID-19 vaccine hesitancy with reasons	-	x	x	x	-
COVID-19 vaccine side effects	-	x	x	x	-
Intent to receive COVID-19 vaccine	-	x	x	x	-
Vaccine perceptions and attitudes	-	x	x	-	-
COVID-19 vaccine information sources	-	-	-	x	-
Body weight and height	-	-	x	x	-
Current and past medical conditions	x	-	-	-	-

CHILD, Canadian Healthy Infant Longitudinal Development; COVID-19, coronavirus disease 2019; x, denotes that question was asked in the survey.

**Table 2.** Questionnaire completion and biological sample collection among 1,462 households participating in the CHILD COVID-19 Add-On Study (n=5,378 children and adults)

Questionnaires	Baseline	Follow-up 1	Follow-up 2	Follow-up 3
Collection period	Jan-Jun, 2021	Aug-Sep, 2021	Oct-Dec, 2021	Jan-Mar, 2022
Completion rate by participant type				
Child self-report	1,643/2,064 (79.6)	987/2,064 (47.8)	880/2,064 (42.6)	794/2,064 (38.5)
Parent on behalf of child	2,336/2,766 (84.4)	1,527/2,766 (55.2)	1,275/2,766 (46.1)	1,202/2,766 (43.5)
Adult self-report	2,308/2,576 (89.6)	1,563/2,576 (60.7)	1,370/2,576 (53.2)	1,252/2,576 (48.6)
Overall completion rate	6,287/7,406 (84.9)	4,077/7,406 (55.0)	3,525/7,406 (47.6)	3,248/7,406 (43.9)
**Biological samples**	**Phase A (n=5,326)**		**Phase B (n=3,219)^1^**	
**Collection period**	**Mar-Sep, 2021**		**Oct-Dec, 2021**	
Sample type	Stool	Blood	Stool	Blood
Children	1,445/2,738 (52.8)	1,462/2,738 (53.4)	823/1,648 (49.9)	847/1,648 (51.4)
Adults	1,506/2,588 (58.2)	1,567/2,588 (60.5)	969/1,571 (61.7)	1,022/1,571 (65.0)
Total	2,951/5,326 (55.4)	3,029/5,326 (56.9)	1,792/3,219 (55.6)	1,869/3,219 (58.1)

Values are presented as number/Number (%). CHILD, Canadian Healthy Infant Longitudinal Development; COVID-19, coronavirus disease 2019.

1 Households who returned biological samples in phase A were invited to participate in phase B.

**Table 3.** Characteristics of CHILD COVID-19 Add-On Study participants at baseline (Jan-Jun, 2021)

Characteristics	Study site				Total
	Vancouver	Edmonton	Manitoba	Toronto	
Households					
Eligible for study	700 (100)	687 (100)	937 (100)	709 (100)	3,033 (100)
Consented for study (% of eligible)	407 (58.1)	308 (44.8)	447 (47.7)	300 (42.3)	1,462 (48.2)
Individuals enrolled (% of total)	1,468 (27.3)	915 (17.0)	1,800 (33.5)	1,195 (22.2)	5,378 (100)
Mothers from original CHILD Study	398	300	442	291	1,431 (26.6)
Fathers from original CHILD Study	292	132	354	237	1,015 (18.9)
Children from original CHILD Study	399	268	441	294	1,402 (26.1)
Siblings	352	203	521	351	1,427 (26.5)
Other household members	27	12	42	22	103 (1.9)
Ancestry (participants, N)^1^	1,458	909	1,779	1,194	5,340
North America (not First Nations)	124 (8.5)	119 (13.1)	185 (10.4)	90 (7.5)	518 (9.7)
First Nations	25 (1.7)	62 (6.8)	178 (10.0)	19 (1.6)	284 (5.3)
UK or Europe	1,012 (69.4)	662 (72.8)	1,332 (74.9)	789 (66.1)	3,795 (71.1)
Central or South America	29 (2.0)	23 (2.5)	44 (2.5)	55 (4.6)	151 (2.8)
Africa	20 (1.4)	10 (1.1)	24 (1.3)	50 (4.2)	104 (1.9)
Middle East	25 (1.7)	13 (1.4)	5 (0.3)	36 (3.0)	79 (1.5)
Asia or Polynesia	251 (17.2)	81 (8.9)	125 (7.0)	221 (18.5)	678 (12.7)
Australia or New Zealand	34 (2.3)	5 (0.6)	6 (0.3)	6 (0.5)	51 (0.9)
Educational attainment (adults, N)	600	378	729	471	2,178
High school or less	14 (2.3)	30 (7.9)	113 (15.5)	29 (6.2)	186 (8.5)
Postsecondary certificate/diploma	113 (18.8)	124 (32.8)	217 (29.8)	48 (10.2)	502 (23.0)
Bachelor’s degree	243 (40.5)	162 (42.8)	268 (36.8)	226 (48.0)	899 (41.3)
Graduate degree	230 (38.3)	62 (16.4)	131 (18.0)	168 (35.7)	591 (27.1)
Occupation (adults, N)	597	375	730	479	2,181
Working	497 (83.2)	303 (80.8)	638 (87.4)	394 (82.2)	1,832 (84.0)
Not working^2^	100 (16.7)	72 (19.2)	92 (12.6)	85 (17.7)	349 (16.0)
Occupation type (adults, N)	563	354	667	458	2,042
Essential worker^3^	142 (25.2)	112 (31.6)	294 (44.1)	80 (17.5)	628 (30.7)
Pandemic impact on work (adults, N)^1^	616	393	762	499	2,270
Moved to remote work	306 (49.7)	148 (37.6)	277 (36.3)	281 (56.3)	1,012 (44.6)
Lost job, permanently	10 (1.6)	12 (3.0)	16 (2.1)	13 (2.6)	51 (2.2)
Lost job, temporarily	42 (6.8)	31 (7.9)	47 (6.2)	28 (5.6)	148 (6.5)
Got new job	31 (5.0)	18 (4.6)	33 (4.3)	19 (3.8)	101 (4.4)
Reduced work hours	82 (13.3)	57 (14.5)	73 (9.6)	52 (10.4)	264 (11.6)
Increased work hours	93 (15.1)	53 (13.5)	122 (16.0)	92 (18.4)	360 (15.8)
Accessed government support^4^	207 (33.6)	151 (38.4)	266 (34.9)	171 (34.3)	795 (35.0)
Educational attainment (children, N)	608	394	796	489	2,287
Homeschool	3 (0.5)	12 (3.0)	21 (2.6)	10 (2.0)	46 (2.0)
Elementary	514 (84.5)	327 (83.0)	576 (72.4)	423 (86.5)	1,840 (80.4)
Junior high	18 (3.0)	32 (8.1)	101 (12.7)	19 (3.9)	170 (7.4)
High school	18 (3.0)	3 (0.8)	27 (3.4)	7 (1.4)	55 (2.4)
Not in school	55 (9.0)	20 (5.1)	71 (8.9)	30 (6.1)	176 (7.7)
Pandemic impact on school (children in school, N)	550	362	704	449	2,065
School closed in March 2020	454 (82.5)	303 (83.7)	626 (88.9)	358 (79.7)	1,741 (84.3)
Online classes offered	445 (98.0)	290 (95.7)	544 (86.9)	319 (89.1)	1,598 (91.8)
Child attended online - fully	369 (82.9)	273 (94.1)	446 (82.0)	277 (86.8)	1,365 (85.4)
Child attended online - partially	72 (16.2)	13 (4.5)	89 (16.4)	41 (12.9)	215 (13.5)
School reopened before Jul 2020	232 (51.1)	18 (5.9)	115 (18.4)	34 (9.5)	399 (22.9)

Values are presented as number (%). CHILD, Canadian Healthy Infant Longitudinal Development; COVID-19, coronavirus disease 2019.

1 Values do not add to 100% because participants could select multiple responses.

2 On leave, unemployed, retired, stay at home parent.

3 Healthcare, delivery worker, store worker, security, building maintenance.

4 Mortgage or lease/rent payment deferral, personal income support ([e.g., Canadian Emergency Response Benefit, Canadian Emergency Student Benefit], employment insurance, business income support [e.g., Canadian Emergency Wage Subsidy], food bank).

## SUPPLEMENTARY MATERIALS

Supplement Material 1. Sociodemographic characteristics of CHILD COVID-19 Add-on Study participants who did vs. did not complete the final follow-up questionnaire.

Supplement Material 2. Comparison of sociodemographic characteristics of CHILD Cohort Study participants to CHILD COVID-19 Add-on Study participants.

Supplement Material 3. Child Self Report

## References

[1] Huang C, Wang Y, Li X, Ren L, Zhao J, Hu Y (2020). Clinical features of patients infected with 2019 novel coronavirus in Wuhan, China. Lancet.

[2] Wu Z, McGoogan JM (2020). Characteristics of and important lessons from the coronavirus disease 2019 (COVID-19) outbreak in China: summary of a report of 72 314 cases from the Chinese Center for Disease Control and Prevention. JAMA.

[3] Udell JA, Behrouzi B, Sivaswamy A, Chu A, Ferreira-Legere LE, Fang J (2022). Clinical risk, sociodemographic factors, and SARS-CoV-2 infection over time in Ontario, Canada. Sci Rep.

[4] Galvão MH, Roncalli AG (2021). Factors associated with increased risk of death from covid-19: a survival analysis based on confirmed cases. Rev Bras Epidemiol.

[5] Centers for Disease Control and Prevention People with certain medical conditions. https://www.cdc.gov/coronavirus/2019-ncov/need-extra-precautions/people-with-medical-conditions.html.

[6] Price-Haywood EG, Burton J, Fort D, Seoane L (2020). Hospitalization and mortality among Black patients and White patients with COVID-19. N Engl J Med.

[7] Morales DR, Conover MM, You SC, Pratt N, Kostka K, DuarteSalles T (2021). Renin-angiotensin system blockers and susceptibility to COVID-19: an international, open science, cohort analysis. Lancet Digit Health.

[8] Smith C, Odd D, Harwood R, Ward J, Linney M, Clark M (2022). Deaths in children and young people in England after SARS-CoV-2 infection during the first pandemic year. Nat Med.

[9] Sinaei R, Pezeshki S, Parvaresh S, Sinaei R (2021). Why COVID-19 is less frequent and severe in children: a narrative review. World J Pediatr.

[10] Syangtan G, Bista S, Dawadi P, Rayamajhee B, Shrestha LB, Tuladhar R (2021). Asymptomatic SARS-CoV-2 carriers: a systematic review and meta-analysis. Front Public Health.

[11] Weiner DL, Balasubramaniam V, Shah SI, Javier JR, Pediatric Policy Council (2020). COVID-19 impact on research, lessons learned from COVID-19 research, implications for pediatric research. Pediatr Res.

[12] Haug N, Geyrhofer L, Londei A, Dervic E, Desvars-Larrive A, Loreto V (2020). Ranking the effectiveness of worldwide COVID-19 government interventions. Nat Hum Behav.

[13] Subbarao P, Anand SS, Becker AB, Befus AD, Brauer M, Brook JR (2015). The Canadian Healthy Infant Longitudinal Development (CHILD) Study: examining developmental origins of allergy and asthma. Thorax.

[14] Harris PA, Taylor R, Thielke R, Payne J, Gonzalez N, Conde JG (2009). Research electronic data capture (REDCap)--a metadata-driven methodology and workflow process for providing translational research informatics support. J Biomed Inform.

[15] Harris PA, Taylor R, Minor BL, Elliott V, Fernandez M, O’Neal L (2019). The REDCap consortium: building an international community of software platform partners. J Biomed Inform.

[16] Cholette F, Mesa C, Harris A, Ellis H, Cachero K, Lacap P (2021). Dried blood spot specimens for SARS-CoV-2 antibody testing: a multi-site, multi-assay comparison. PLoS One.

[17] World Health Organization (2021). COVID-19 symptoms and severity. https://www.who.int/westernpacific/emergencies/covid-19/information/asymptomatic-covid-19.

