# Supplementary material for: Cohort profile: investigating SARS-CoV-2 infection and the health and psychosocial impact of the COVID-19 pandemic in the Canadian CHILD Cohort
Source: Epidemiol Health. 2023 Oct 13;45:e2023091. doi: 10.4178/epih.e2023091 (PMC10867514; doi:10.4178/epih.e2023091)
Supplement: Supplement Material 1. — Sociodemographic characteristics of CHILD COVID-19 Add-on Study participants who did vs. did not complete the final follow-up questionnaire. [file epih-45-e2023091-Supplementary-1.pdf]

Table S1. Sociodemographic characteristics of CHILD COVID-19 Add-on Study participants who did vs. did not complete the final follow-up questionnaire.

	Enrolled and completed final questionnaire	Enrolled but did not complete final questionnaire	Chi-square *p*-value
**Total participants, N**	**2388**	**2256**	
**Participants**			
Adults	1216 (51)	1065 (47)	0.01*
Children	1172 (49)	1191 (53)	
**Ancestry**†	**2387**	**2232**	
North American (not First Nation)	252 (11)	266 (12)	0.16
First Nation	138 (6)	146 (7)	0.31
UK or Europe	1985 (83)	1810 (81)	0.07
Central or South America	57 (2)	94 (4)	0.001*
Africa	41 (2)	63 (3)	0.02*
Middle East	21 (1)	58 (3)	<0.001*
Asia or Polynesia	394 (17)	284 (13)	<0.001*
Australia or New Zealand	29 (1)	22 (1)	0.55
**Education attainment (adults, N)**	**1197**	**992**	
High school or less	85 (7)	101 (10)	<0.001*
Post-secondary certificate/diploma	268 (22)	237 (24)	
Bachelor's degree	510 (43)	392 (40)	
Graduate degree	334 (28)	262 (26)	
**Occupation (adults, N)**	**1164**	**975**	
Working	990 (85)	842 (86)	0.28
Not Working[1]	174 (15)	133 (14)	
**Occupation type (adults, N)**	**1216**	**1065**	
Essential worker[2]	341 (28)	295 (28)	0.25
**Pandemic impact on work (adults, N)**†	**1215**	**1045**	
Moved to remote work	566 (47)	446 (43)	0.01*
Lost job, permanently	21 (2)	30 (3)	0.05*
Lost job, temporarily	74 (6)	74 (7)	0.15
Got new job	49 (4)	52 (5)	0.14
Reduced work hours	135 (11)	129 (12)	0.17
Increased work hours	186 (15)	174 (17)	0.18
Increased risk of COVID from job	288 (24)	246 (23)	0.17
Accessed government support[3]	399 (33)	396 (37)	0.02*
**Educational attainment (children, N)**	**1170**	**1126**	
Home school	29 (3)	17 (1)	0.05
Elementary school	943 (80)	897 (80)	
Junior high	80 (7)	91 (8)	
High school	23 (2)	40 (4)	
Not in school	95 (8)	81 (7)	
**Pandemic impact on school (children in school, N)**	**1046**	**1028**	
School closed in March 2020	878 (84)	863 (84)	0.50
Of schools that closed,			
Online classes offered	814 (93)	784 (91)	0.13
Online classes attended - fully	708 (88)	657 (85)	0.14
Online classes attended - partially	96 (12)	119 (15)	0.06
School reopened before July 2020	206 (24)	200 (23)	0.82

Values are n (%) for participants with non-missing data for each variable. Values are derived from the baseline questionnaires completed between Jan – Jun 2021. †Values do not add to 100% because participants could select multiple responses. * $p < 0.05$.
[1]On leave, unemployed, retired, stay at home parent. [2]Healthcare, delivery worker, store worker, security, building maintenance.
[3]Mortgage or Lease/ Rent Payment deferral, Personal Income Support (e.g., Canadian Emergency Response Benefit (CERB), Canadian Emergency Student Benefit (CESB), Employment Insurance (EI)), Business Income Support (e.g., Canadian Emergency Wage Subsidy (CEWS)), Food Bank.
