# Supplementary material for: Cohort profile: investigating SARS-CoV-2 infection and the health and psychosocial impact of the COVID-19 pandemic in the Canadian CHILD Cohort
Source: Epidemiol Health. 2023 Oct 13;45:e2023091. doi: 10.4178/epih.e2023091 (PMC10867514; doi:10.4178/epih.e2023091)
Supplement: Supplement Material 2. — Comparison of sociodemographic characteristics of CHILD Cohort Study participants to CHILD COVID-19 Add-on Study participants. [file epih-45-e2023091-Supplementary-2.pdf]

Table S2. Comparison of sociodemographic characteristics of CHILD Cohort Study participants to CHILD COVID-19 Add-on Study participants.

Variable	All CHILD households†	CHILD households involved in COVID-19 Study	CHILD households not involved in COVID-19 Study	Chi-square *p*-value‡
**Total participants, N**	**3263**	**1386**	**1877**	
**Study centre at CHILD enrollment**				
Vancouver	731 (21)	378 (27)	353 (19)	<0.001*
Edmonton	767 (24)	296 (21)	471 (25)	
Manitoba	994 (31)	427 (31)	567 (30)	
Toronto	771 (24)	285 (21)	486 (26)	
**Parents' marital status (participants, N)**	**3184**	**1379**	**1805**	
Single	169 (5)	52 (4)	117 (7)	0.003*
Married/common law	2989 (94)	1316 (95)	1673 (92)	
Divorced/separated/widowed	26 (1)	11 (1)	15 (1)	
**Mother's race (participants, N)**	**3207**	**1379**	**1828**	
European/White	2339 (73)	1067 (78)	1272 (70)	<0.001*
First Nations	141 (4)	47 (3)	94 (5)	
Asian	505 (16)	191 (14)	314 (17)	
Other	222 (7)	74 (5)	148 (8)	
**Father's race (participants, N)**	**3218**	**1383**	**1835**	
European/White	2377 (74)	1065 (78)	1312 (71)	<0.001*
First Nations	128 (4)	58 (4)	70 (4)	
Asian	442 (14)	171 (12)	271 (15)	
Other	271 (8)	89 (6)	182 (10)	
**Mother's age (Mean ± SD)**	37.9 ± 4.5	38.1 ± 4.1	37.6 ± 4.8	0.01*
**Father's age (Mean ± SD)**	39.8 ± 5.3	39.9 ± 5.0	39.7 ± 5.7	0.38
**Mother's education (participants, N)**	**3136**	**1361**	**1775**	
High school or less	276 (9)	59 (4)	217 (12)	<0.001*
Postsecondary certificate/diploma	655 (21)	283 (21)	372 (21)	
Bachelor's degree	1605 (51)	725 (53)	880 (50)	
Graduate degree	600 (19)	294 (22)	306 (17)	
**Father's education (participants, N)**	**3104**	**1347**	**1757**	
High school or less	475 (15)	145 (11)	330 (19)	<0.001*
Postsecondary certificate/ diploma	813 (26)	345 (26)	468 (26)	
Bachelor's degree	1322 (43)	639 (47)	683 (39)	
Graduate degree	494 (16)	218 (16)	276 (16)	
**Household income (households, N)**	**2890**	**1288**	**1602**	
< $30,000	127 (4)	31 (2)	96 (6)	<0.001*
$30,000-$59,999	352 (12)	110 (9)	242 (15)	
$60,000-$99,999	667 (23)	298 (23)	369 (23)	
$100,000-$149,999	822 (29)	388 (30)	434 (27)	
≥ $150,000	922 (32)	461 (36)	461 (29)	
**Household size (households, N)**	**3121**	**1373**	**1748**	
1-3	1046 (34)	335 (24)	711 (41)	<0.001*
4-6	1965 (63)	990 (72)	975 (56)	
>6	110 (3)	48 (4)	62 (3)	

Values are n (%) or mean ± standard deviation, for households with non-missing data for each variable. Values reflect most recently available data up to 5-year CHILD study visit (completed in 2018-2020), unless otherwise stated. "Enrollment" refers to the original CHILD cohort study, undertaken in 2009-12.

* $p < 0.05$. †General cohort (excludes Vanguard cohort of the CHILD study). ‡Chi-square (categorical data) and t-test (continuous data) *p*-value for comparison of CHILD participants involved vs not involved in CHILD COVID-19 Add-on Study.
